# Awareness of the CDC “Heads Up!” to Youth Sports Campaign among Pediatric Sports Coaches: A Pilot Survey Study

**DOI:** 10.7759/cureus.755

**Published:** 2016-08-29

**Authors:** Thor S Stead, Vaibhav Rastogi, Vishnumurthy S Hedna, Latha Ganti

**Affiliations:** 1 College of Mathematics, University of Central Florida; 2 Department of Medicine, University of Central Florida; 3 Department of Neurology, University of New Mexico; 4 Clinical Sciences, University of Central Florida

**Keywords:** concussion awareness, concussion, sports medicine, centers for disease control

## Abstract

Objectives:

This study sought to: 1) estimate the penetrance (in terms of youth coaches being aware of them) of the CDC “Heads Up!” guidelines, 2) determine whether these guidelines changed the coaches’ practice, and 3) understand whether these guidelines resulted in a perceived decrease in the number of concussions.

Methods:

This was a cross-sectional survey of pediatric sports coaches in the United States designed to assess the impact of the CDC "Heads Up!" guidelines. “Heads Up!” Concussion in Youth Sports is a free, online course available to coaches, parents, and others helping to keep athletes safe from concussions. The “Heads Up!” fact sheet provides important information on preventing, recognizing, and responding to a concussion.

Results:

Half the cohort had heard of the CDC “Heads Up!” campaign. Fifty-five percent of the cohort thought that pediatric concussions in youth sports was a “big deal” (rated on a Likert scale from 1-10). Coaches who were also parents (58%) were significantly more likely to have heard of the campaign (P=0.0032, 95% CI=0.1153-0.5513). Having heard of the “Heads Up!” campaign was significantly associated with how important coaches thought pediatric concussions are (P=0.0133, 95% CI=0.0590-0.4960), as was higher income of the coaches (P=0.0100), and this was significantly correlated with the coach being more likely to call the athlete’s parent at injury (P=0.0030, 95% CI=0.1160-0.5471). Coaches of football/soccer were significantly more likely to think pediatric concussions were a “big deal” (P=0.0021,95% CI=0.1374-0.5947). More than a third of coaches 35% reported that the “Heads Up!” campaign decreased the number of concussions on their team.

## Introduction

A concussion is defined as the complex pathophysiological processing involving the brain that occurs as a result of biomechanical forces [1]. These forces can be transmitted by a direct blow to the head, face or neck or indirectly from any other body part to the brain. It usually causes transient symptoms that have a rapid onset and is not associated with any structural changes. Due to its functional nature, neuroimaging is often negative. The typical presentation includes confusion and amnesia after a trauma. The post-concussive syndrome can ensue after a concussion and patients may experience loss of consciousness, headache, nausea, vomiting, dizziness, visual disturbances, alterations in sleep patterns, and behavioral modification, among others [2]. Sports can have a significant causative association with concussions. There are 4,000,000 sports-related concussions in the United States annually [3]. A recent study reported practice and game concussion incidence rate in eight to 12-year-olds to be 0.24 and 6.16 per 1000 athlete-exposures [4]. Football in males and basketball and soccer in females are considered maximum risk high school sports. Rugby, ice hockey, and lacrosse are other sports with high risk of concussion. It has been noted that the incidence rate in females is greater than males [5]. To help ensure the health and safety of young athletes, the CDC developed the “Heads Up!” Concussion in Youth Sports [6] initiative to offer information about concussions to coaches, parents, and athletes involved in youth sports. “Heads Up!” Concussion in Youth Sports is a free, online course available to coaches, parents, and others, helping to keep athletes safe from concussion. The “HEADS UP” fact sheet [7] provides important information on preventing, recognizing, and responding to a concussion. The objectives of this study were to: 1) estimate the penetrance (in terms of youth coaches being aware of them) of the CDC “Heads Up!” guidelines, 2) determine whether these guidelines changed coaches’ practice, and 3) understand whether these guidelines resulted in a perceived decrease in number of concussions.

## Materials and methods

A cross-sectional survey of pediatric sports coaches in the United States was conducted using Google Consumer Survey methodology to assess how many had heard of this campaign, and whether it affected their practice. Google Consumer Surveys [8] show questions across a network of premium online news, reference, and entertainment sites, where they get embedded directly into content, as well as through a mobile app, Google Opinion Rewards. On the web, users answer questions in exchange for access to that content, an alternative to subscribing or upgrading. The user's gender, age, and geographic location are inferred based on anonymous browsing history and IP address. On mobile, users answer questions in exchange for credits for books, music, and apps and users answer demographic questions when first downloading the app. Using this data, Google Consumer Surveys builds a representative sample of thousands of respondents. This Google consumer survey was administered in such a way as to garner a validated, representative sample regarding gender, location, and basic demographics of coaches of youth sports. However, the responses were anonymous, and no protected health information was collected in an identifiable manner. The demographics were collected by the Google survey team, and the specifics were not known to the researcher. Thus, informed consent was not applicable. The background questions targeted the type of sport coached, whether the coach was him/herself a parent, how relevant a concussion was in the sport they coached, and the actual number of concussions they saw on their team in a given season. The CDC knowledge questions asked whether the coach had heard of the “Heads Up!” campaign, if and how they implemented it in their practice, and whether or not it impacted the actual number of concussions. Responses were analyzed using JMP 12.0 for the Macintosh (SAS Institute, Cary, NC, USA). As this was a voluntary, anonymous survey of pediatric sports coaches, the work is in compliance with the Declaration of Helsinki [9] and exempt from additional institutional board review.

## Results

**Figure 1:**
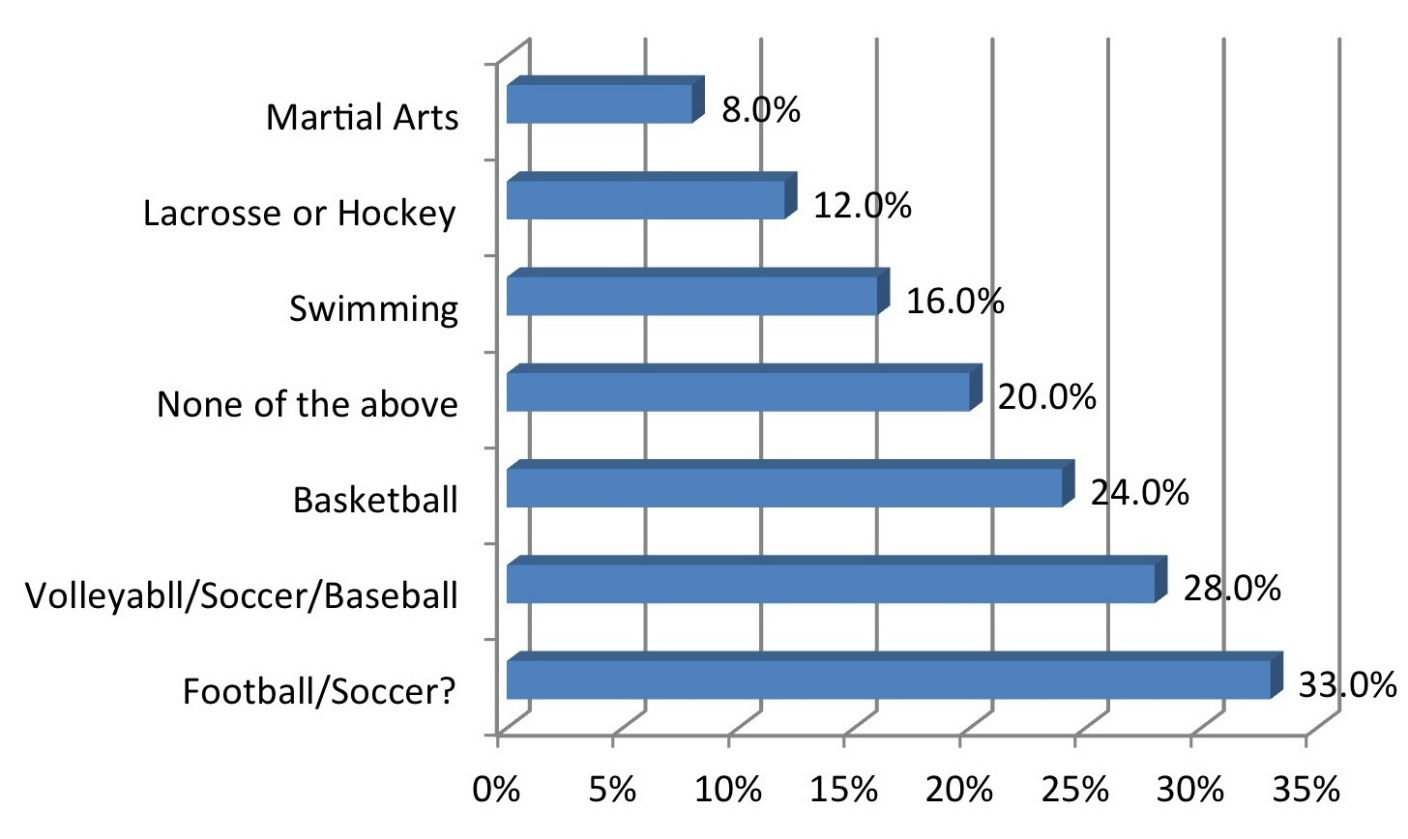
Which sport do you coach?

The cohort comprised 56% male, 17% of them within an age range of 18-24 years, 13% within 25-34 years, 29% within 35-44 years, 21% within 45-54 years, 12% within 55-64 years, and 8% with age 65+. Regarding the United States regional representation, 29% were from the Midwest, 21% from the Northeast, 27% from the South, and 23% from the West. The majority identified their location as suburban (54%) while 19% reported rural, and 27% urban. Income ranges of the coaches ranged from $0-24K (8%), $25-49k (55%), $50-74K (28%), $75-99k (6%), and over $100k (3%). Regarding the sports coached (Figure 1), 32% reported football, 8% martial arts, 12% lacrosse or hockey, 24% basketball, 28% volleyball, baseball or softball, and 17% swimming.

**Figure 2:**
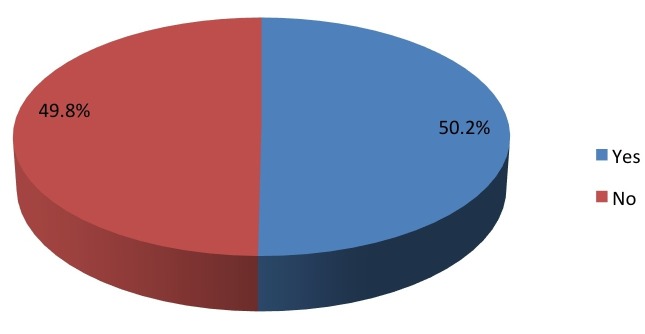
Have you ever heard of the Center for Disease Control "Heads Up!" Campaign?

A little over half the cohort (50.2%) had heard of the CDC “Heads Up!” campaign (Figure 2).

**Figure 3:**
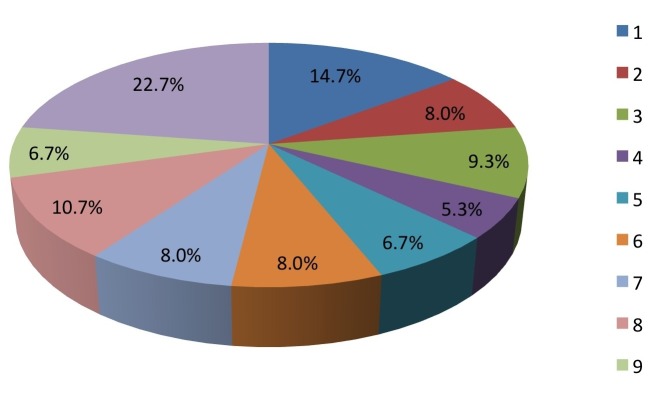
On a scale from 1-10, how “big a deal” do you feel concussion is in your sport/sports? (1=not a big deal, 10=huge deal)

Similarly, 55% thought pediatric concussions in youth sports was a “big deal” (rated on a Likert scale from 1-10) (Figure 3).

**Figure 4:**
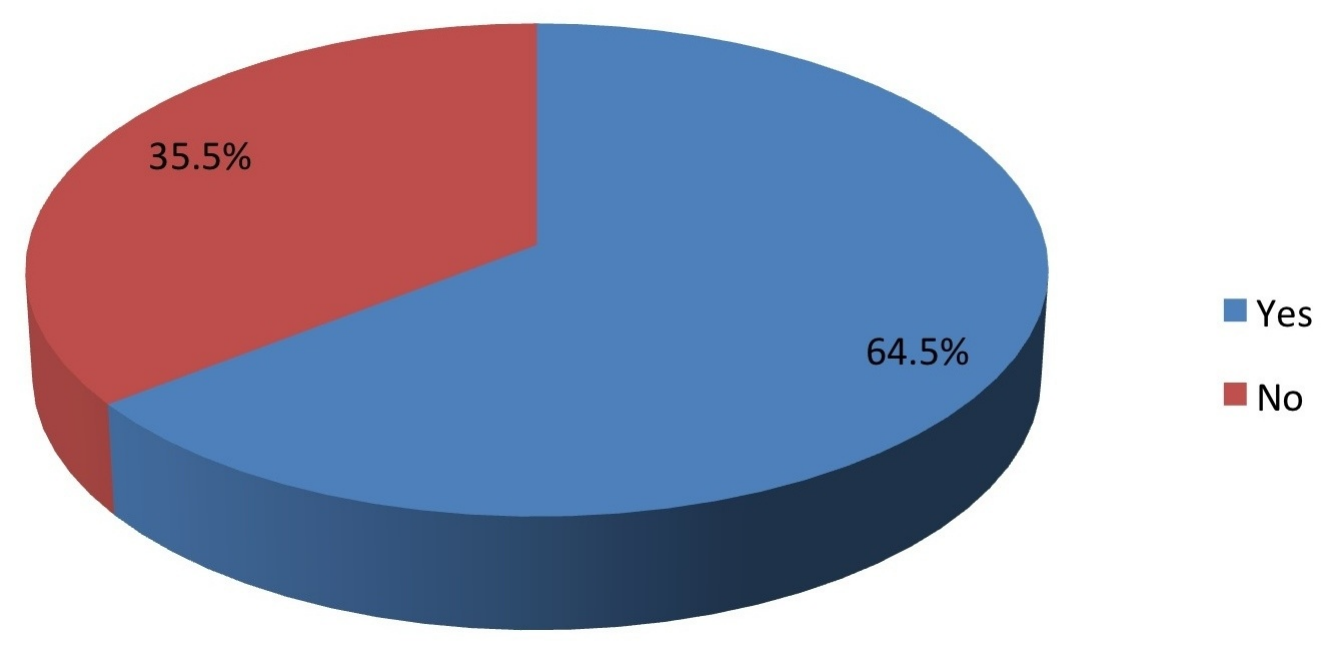
Are you a parent?

Coaches who were also parents (58%, Figure 4) were significantly more likely to have heard of the campaign (P=0.0035).

**Figure 5:**
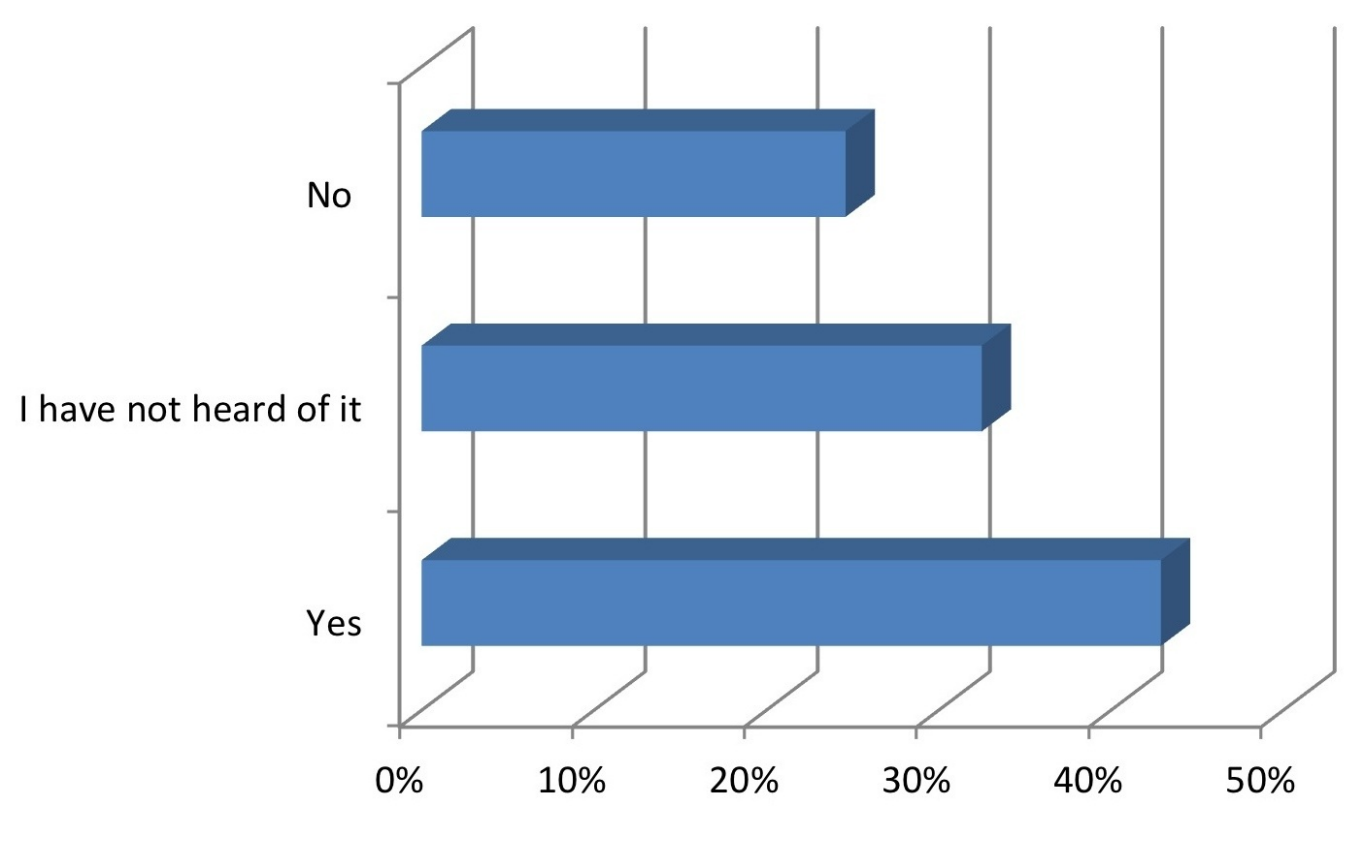
Do you follow "Heads Up Concussion" guidelines on the return to play of an injured athlete?

**Figure 6:**
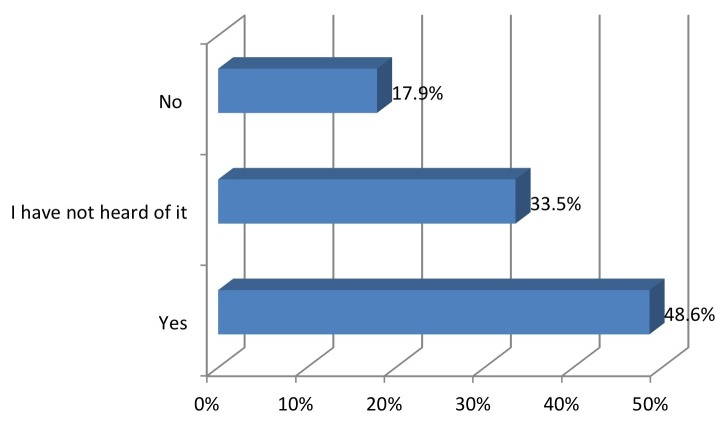
If you use the "Heads Up Concussion" guidelines or have heard of it, do you teach it to your athletes?

**Figure 7:**
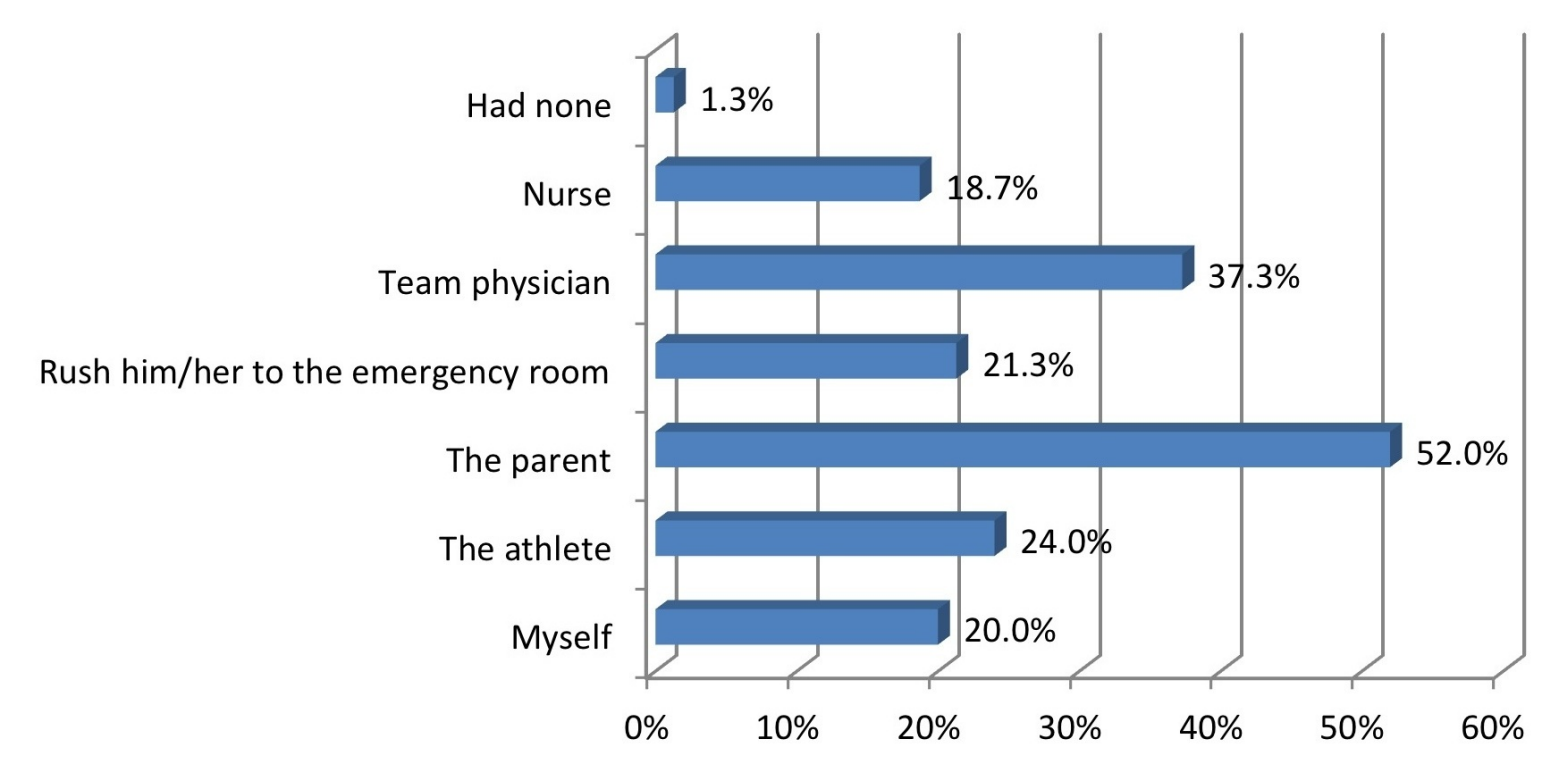
Whom do you usually consult immediately following an athlete's concussion?

Higher ratings of the importance of pediatric concussions were significantly correlated with coaches having heard of the “Heads Up!” program (P=0.0084). The knowledge of the program was significantly correlated with the coach being more likely to call the athlete’s parent at injury (P=0.0033, Fisher’s exact test; P=0.0034, Pearson correlation) (Figures 5-7). 

**Figure 8:**
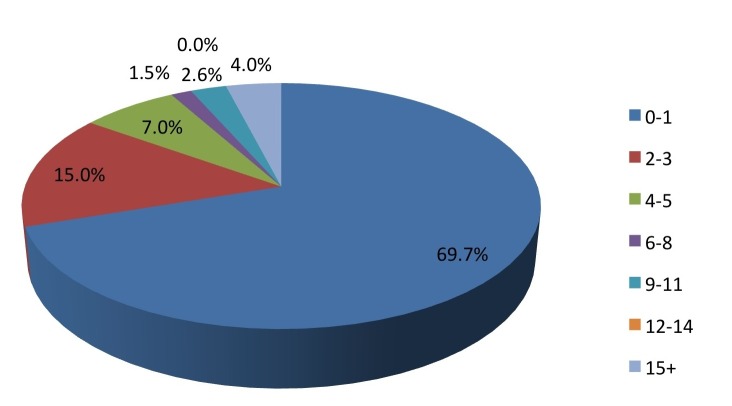
On average, how many concussions do you have per season?

Of all the sports, coaches of football/soccer were significantly more likely to think pediatric concussions were a “big deal” (P=0.0022). The number of concussions per season ranged from 0-1 (69%), 2-3 (15%), 4-5 (6%), 6-14 (5%), and >15 (4%) (Figure 8). 

**Figure 9:**
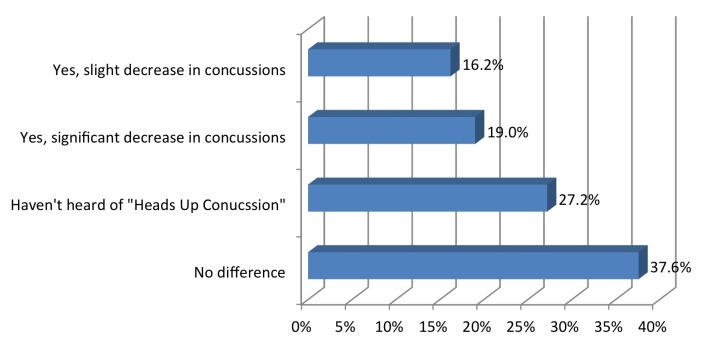
Has "Heads Up Concussion" decreased the number of concussions per season? What was the magnitude of the change?

A total of 35% reported that the “Heads Up!” campaign decreased the number of concussions on their team (Figure 9).

## Discussion

Despite a tremendous effort to educate our school coaches about the dangers of pediatric concussions with a freely accessible CDC “Heads Up!” toolkit only about half of coaches from a cross-sectional survey of youth sports coaches across the United States are aware of this resource. This penetration is similar to that seen in pediatric emergency departments, where only 12% of discharged children received a recommendation of cognitive rest [10]. It is also in agreement with a study of parents of five to 15-year-olds participating in tackle football that showed that only 13% could correctly answer questions based on the CDC “Heads Up!” Concussion in Youth Sports Quiz [11]. A survey of members of the National Association of School Nurses showed only 53% of schools have guidelines to assist students when returning to school after a concussion [12]. Thus, the current study underscores the need to continue to educate our coaches, and all personnel who come into contact with potential youth concussions. The most significant finding of the current study is that knowledge of these guidelines results in fewer concussions overall.

Concussions pose a major public health problem. The estimated incidence of sports-related concussions in the USA ranges from 3,00,000 to 3.8 million annually [13], and this is likely an underestimate as sports-related concussions frequently go undetected due to a lack of recognition of symptoms or intentional underreporting of symptoms [14,15]. There are both the acute primary sequelae which include a headache, dizziness, nausea, and imbalance, as well as the secondary sequelae termed as second impact syndrome. Secondary impact syndrome occurs from a premature return to sports, after which subsequent, even relatively minor, contact affects the brain in a more vulnerable state [15]. The impact can be as serious as increased intracranial pressure, brainstem herniation, and death [17]. As a result of these severe injury sequelae, and the sheer volume of the occurrence of these instances, concussions truly are a public health emergency [16]. Management of concussions includes cognitive rest, graduated activity, and strict adherence to return-to-play guidelines [18]. Even though all of this information is known and published, the CDC estimates that at least 15% of high school athletes diagnosed with concussion fail to comply with recommended return-to-play guidelines. Thus, the education of athletes, coaches, parents, and physicians in improving recognition of potential concussive injuries is imperative [19].

## Conclusions

Awareness of the CDC "Heads Up" Concussion guidelines is significantly associated with a decrease in the total number of concussions during the season. This finding highlights the importance of such campaigns. In light of this, one should consider whether the CDC Heads up toolkit, or other similar guideline usages should be made mandatory, as any intervention to decrease the overall rate of concussions is a crucial preventative health initiative. Also, awareness of the coaches on the vital role they play in the prevention of this important public health problem is imperative.
